# An Action for Mal-Practice

**Published:** 1898-05-14

**Authors:** 


### AN ACTION FOR MAL-PRACTICE.

The interest of the sort of accident above-mentioned—for surely it may sometimes be properly called an accident—is driven home to us all the more by a recent suit for mal-practice brought against a New York medical man, in regard to a case in which a piece of gauze was removed from the bottom a mural abscess left after the performance of cœliotomy. The case was a curious one. First, vagino-fixation of the uterus was performed; after this an abscess occurred, and was drained through the posterior vaginal fornix. A small sinus, however, was left, so an attempt was made to find the ligature, which was supposed to be the cause. This, however, was unsuccessful. Then it was decided to open the abdomen and remove the ligature. This was done with considerable difficulty, for the patient was very stout.

After this operation a mural abscess formed, which was treated by irrigation and packing, but refused to heal. Finally, she went into a public institution, where, on the sinus being enlarged, a piece of gauze "the size of the thumb" was removed from the bottom of the wound, which then closed. On this an action for damages, fixed at 10,000 dollars, was brought against the surgeon, on the ground that the piece of gauze had been carelessly left behind during the performance of cœliotomy. There was, however, no clear evidence that the surgeon who did the operation had introduced the gauze, for the wound had during several weeks been dressed by other parties, often by nurses.

The verdict was for the defendant, but the case well illustrates the risks which are run by operating surgeons when anything occurs in their work which can be twisted into a semblance of carelessness.

